# Crystal structure of the solid solution Ba_8.35_Pb_0.65_(B_3_O_6_)_6_

**DOI:** 10.1107/S2056989017001864

**Published:** 2017-02-14

**Authors:** Wenwu Zhao

**Affiliations:** a Department of Environmental and Chemical Engineering, Tangshan College, 38 North HuaYan Road, Tangshan 063000, Hebei, People’s Republic of China

**Keywords:** crystal structure, borate, spontaneous nucleation, solid solution

## Abstract

Single crystals of lead barium borate, Ba_8.35_Pb_0.65_(B_3_O_6_)_6_, octabarium lead(II) hexa­kis­(triborate), have been obtained by spontaneous nucleation from a high-temperature melt. Its three-dimensional structure is constructed on the basis of a BaO_9_ polyhedron, a (Pb/Ba)O_6_ octa­hedron (occupancy ratio Pb:Ba = 0.216:0.784) and a condensed B_3_O_6_ ring anion. In the crystal, the planar B_3_O_6_ anions are stacked in an alternating fashion with Ba and (Pb/Ba) atoms along [001]. A comparison is made with the structures of related solid solutions in the system Ba/Pb/B/O.

## Abstract 2

The fundamental building units of Ba_8.35_Pb_0.65_(B_3_O_6_)_6_ are isolated planar B_3_O_6_ anionic groups, which are distributed layer upon layer perpendicular to [001], with (Pb/Ba) and Ba sites alternately located between the B_3_O_6_ sheets.

## Chemical context   

The study of inorganic borates is motivated by their possible non-linear optical properties, transparency in a wide range of wavelengths, high laser-damage tolerance, piezoelectricity and luminescent and other useful properties for technical applications of the respective compounds. For example, *β*-BaB_2_O_4_ (Chen *et al.*, 1985 ▸), LiB_3_O_5_ (Chen *et al.*, 1989 ▸), CsB_3_O_5_ (Sasaki *et al.*, 2000 ▸), Sr_2_Be_2_B_2_O_7_ (Chen *et al.*, 1995 ▸), K_5_Ba_10_(BO_3_)_8_F (Liu *et al.*, 2016 ▸), PbB_4_O_7_ (Bartwal *et al.*, 2001 ▸), Pb_2_B_5_O_9_ *X* (*X* = Cl, Br, I) (Huang *et al.*, 2010 ▸) or Ba_3_Sr_4_(BO_3_)_3_F_5_ (Zhang *et al.*, 2009 ▸) have been studied because of their second-order non-linear optical behavior. Among inorganic borates synthesized and characterized over the past decades, some lead(II) borates show comprehensive applications. These features are associated with the highly asymmetric stereochemistry typical for a lead(II) atom due to the stereoactivity of the 6*s* ^2^ lone pair (Zhang *et al.*, 2016 ▸; Mutailipu *et al.*, 2016 ▸). Accordingly, numerous studies have been devoted to this family of compounds. Some lead borates are particularly attractive because of their high second-harmonic generation (SHG) response (Wu *et al.*, 2012 ▸; Dong *et al.*, 2015 ▸; Jing *et al.*, 2015 ▸) or large birefringence (Liu *et al.*, 2015 ▸).

In this communication, we report on the synthesis and crystal structure of the solid solution Ba_8.35_Pb_0.65_(B_3_O_6_)_6_.

## Structural commentary   

**Figure 1:**
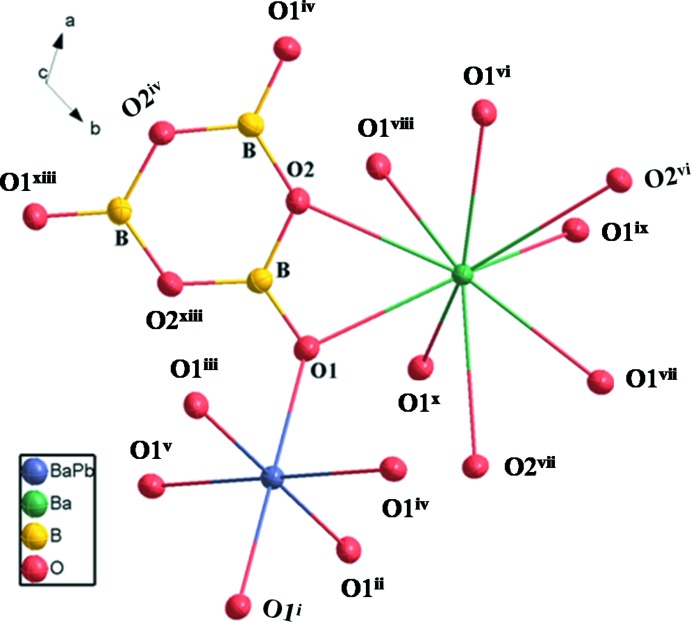
The principal building units in the crystal structure of the title compound. Displacement ellipsoids are drawn at the 50% probability level. [Symmetry codes: (i) −*x*, −*y*, −*z*; (ii) −*y*, *x* − *y*, *z*; (iii) *y*, −*x* + *y*, −*z*; (iv) *x* − *y*, *x*, −*z*; (v) −*x* + *y*, −*x*, *z*; (vi) −*y* + 1, *x* − *y*, *z*; (vii) −*x* + *y* + 1, −*x* + 1, *z*; (viii) *y* + 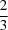, −*x* + *y* + 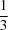, −*z* + 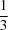; (ix) *x* − *y* + 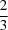, *x* + 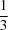, −*z* + 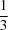; (x) −*x* + 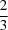, −*y* + 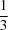, −*z* + 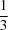; (xiii) −*y*, *x* − *y* − 1*, z*; (xiv) −*x* + *y* + 1, −*x*, *z*.]

**Figure 2:**
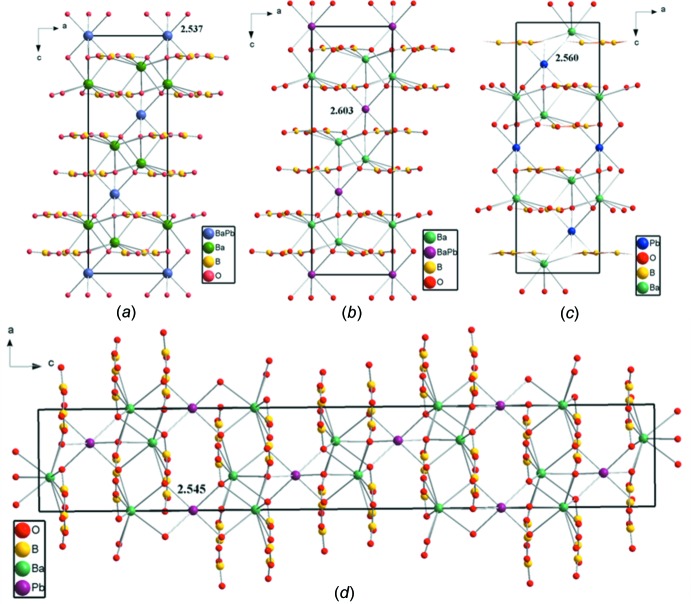
The crystal structures of related solid solutions in the system Ba/Pb/B/O viewed down [010]: (*a*) Ba_8.35_Pb_0.65_(B_3_O_6_)_6_; (*b*) Ba_7.87_Pb_1.13_(B_3_O_6_)_6_ (Wu *et al.*, 2012 ▸); (*c*) Ba_2_Pb(B_3_O_6_)_2_ (Li *et al.*, 2014 ▸); (*d*) Ba_2_Pb(B_3_O_6_)_2_ (Tang *et al.*, 2015 ▸). The numbers indicate the bond lengths (Å) of the PbO_6_ or (Ba/Pb)O_6_ octa­hedra.

The crystal structure of Ba_8.35_Pb_0.65_(B_3_O_6_)_6_ is based on a Ba2O_9_ polyhedron, a (Pb/Ba1)O_6_ polyhedron and a condensed B_3_O_6_ anion, as shown in Fig. 1 ▸. The planar B_3_O_6_ anions (point group symmetry 3.) are isolated from each other and distributed layer upon layer perpendicular to [001]. The occupationally disordered (Pb/Ba)1 site (occupancy ratio Pb:Ba = 0.216:0.784) and the Ba2 site are located alternately between the B_3_O_6_ sheets in (Pb/Ba)1 and Ba2 layers, as shown in Fig. 2 ▸ *a*. The B atom is bound to one O1 atom and two O2 atoms to from a BO_3_ triangle. Three BO_3_ triangles are condensed through vertex-sharing to build a planar and cyclic B_3_O_6_ unit. The B—O bond lengths vary from 1.318 (5) to 1.406 (5) Å (Table 1 ▸), and the O—B—O angles are between 116.8 (4) and 122.6 (4)°.

**Figure 3:**
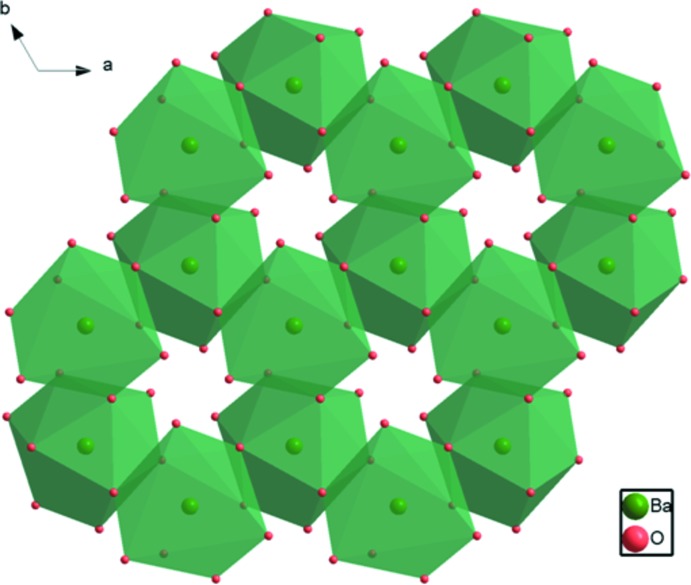
The formation of a corrugated layer of Ba2O_9_ polyhedra in the crystal structure of Ba_8.35_Pb_0.65_(B_3_O_6_)_6_ viewed down [001].

The Ba2 atom (site symmetry 3.) is coordinated by nine O atoms. The Ba—O bond lengths of the Ba2O_9_ polyhedron range from 2.766 (3) to 3.030 (3) Å, with a mean distance of 2.869 Å (Table 1 ▸). A similar environment for Ba is observed in the crystal structures of Na_3_Ba_2_(B_3_O_6_)_2_F (Zhang *et al.*, 2015 ▸), PbBa_2_(B_3_O_6_)_2_ (Li *et al.*, 2014 ▸) and α-BBO (Wu *et al.*, 2002 ▸). Each of the Ba2O_9_ polyhedra shares edges with adjacent Ba2O_9_ polyhedra to form six-membered rings that are arranged in corrugated layers extending parallel to (001) (Fig. 3 ▸). The (Pb/Ba)1 site (site symmetry 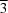.) is surrounded by six O atoms; the corresponding (Pb/Ba)1O_6_ octa­hedra are isolated from each other. The six (Pb/Ba1)—O bonds have an identical length of 2.537 (3) Å (Table 1 ▸, Fig. 2 ▸ *a*). In comparison with the *M*2 site, the *M*1 site has a more narrow coordin­ation environment which seems to be the reason why Pb atoms exclusively substitute Ba atoms at the latter position due to their smaller ionic radius.

## Comparison with the structures of related solid solutions   

It is inter­esting to compare the structure of Ba_8.35_Pb_0.65_(B_3_O_6_)_6_ with those of the related solid solutions Ba_7.87_Pb_1.13_(B_3_O_6_)_6_ (Wu *et al.*, 2012 ▸) and Ba_2_Pb(B_3_O_6_)_2_ (Li *et al.*, 2014 ▸; Tang *et al.*, 2015 ▸). Whereas the title compound Ba_8.35_Pb_0.65_(B_3_O_6_)_6_ crystallizes in space group *R* 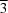, Ba_7.87_Pb_1.13_(B_3_O_6_)_6_ was solved and refined in space group *R*32 on the basis of single crystal X-ray diffraction data (Wu *et al.*, 2012 ▸); the lattice parameters of both compounds are very similar. Ba_2_Pb(B_3_O_6_)_2_ on the other hand was reported to crystallize either in space group *R* 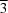 with lattice parameters in the same range as the previous two structures (single crystal X-ray diffraction data; Li *et al.*, 2014 ▸) or in space group *R* 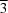 *c* with a doubled *c* axis in comparison with the other structures (powder X-ray diffraction data using the Rietveld method; Tang *et al.*, 2015 ▸). All four crystal structures are characterized by an alternating stacking of cationic and anionic (001) layers along [001], as shown in Fig. 2 ▸. In each case, the Ba site is coordinated by nine O atoms to form BaO_9_ polyhedra, and the Pb or the (Pb/Ba) site is coordinated by six O atoms to form distorted PbO_6_ octa­hedra [in Ba_2_Pb(B_3_O_6_)_2_] or (Pb/Ba)O_6_ octa­hedra [in Ba_8.35_Pb_0.65_(B_3_O_6_)_6_ and Ba_7.87_Pb_1.13_(B_3_O_6_)_6_].

**Figure 4:**
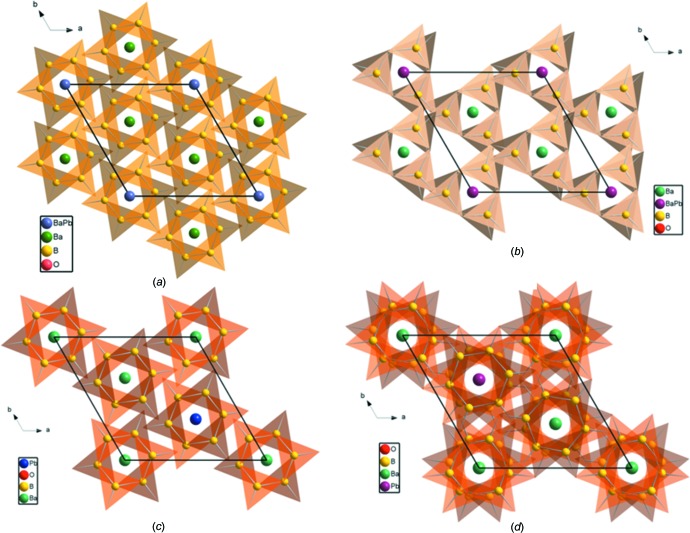
The arrangement of B_3_O_6_ groups along the [001] direction in the different solid solutions: (*a*) Ba_8.35_Pb_0.65_(B_3_O_6_)_6_; (*b*) Ba_7.87_Pb_1.13_(B_3_O_6_)_6_ (Wu *et al.*, 2012 ▸); (*c*) Ba_2_Pb(B_3_O_6_)_2_ (Li *et al.*, 2014 ▸); (*d*) Ba_2_Pb(B_3_O_6_)_2_ (Tang *et al.*, 2015 ▸).

The arrangement of the planar B_3_O_6_ rings in the crystal structures is a determining factor in whether a non-centrosymmetric or a centrosymmetric structure is obtained. In Ba_7.87_Pb_1.13_(B_3_O_6_)_6_ (Wu *et al.*, 2012 ▸), the rings are aligned in a chiral arrangement (Fig. 4 ▸ *b*), responsible for the SHG effect. In Ba_2_Pb(B_3_O_6_)_2_ (Li *et al.*, 2014 ▸), the B_3_O_6_ rings are parallel to each other, distributed layer upon layer along [001], and the B_3_O_6_ rings in neighbouring layers point in exactly opposite directions (Fig. 4 ▸ *c*), just like in the title compound (Fig. 4 ▸ *a*). In the Ba_2_Pb(B_3_O_6_)_2_ structure with doubled volume (Tang *et al.*, 2015 ▸), all of the B_3_O_6_ rings are parallel to (001), and the B_3_O_6_ rings in two neighbouring layers are rotated slightly relative to each other (Fig. 4 ▸ *d*).

## Synthesis and crystallization   

Suitable crystals of the solid solution Ba_8.35_Pb_0.65_(B_3_O_6_)_6_ were obtained by spontaneous nucleation from a high-temperature melt mixture originating from PbO, H_3_BO_3_ and BaF_2_ in molar ratios of 4:5:1. The starting materials were weighed and melted in a platinum crucible in several batches. The crucible position was fixed at the centre of a resistance-heated furnace. The temperature of the furnace was controlled within 0.1–1 K by an Al-708P controller and a Pt/Pt–Rh thermocouple. The temperature was raised by about 50 K h^−1^ to 50 K above the melting point and held for 15 h to ensure a homogenous mixture of the solution. After cooling down the furnace to 1073 K, a slow cooling rate of 5 K d^−1^, was applied, followed by cooling to room temperature at 20 K h^−1^. Colorless crystals in the millimetre range were obtained.

## Refinement   

Crystal data, data collection and structure refinement details are summarized in Table 2 ▸. From the two large cation positions in the structure (Wyckoff positions 3*a* and 6*c*), only those of *M*1 at 3*a* are occupationally disordered by Ba and Pb atoms. Refinement of the occupancy of Ba:Pb at this site under consideration of EXYZ and EADP commands (Sheldrick, 2008 ▸) resulted in a 21.6 (7)% occupancy of Pb. The highest peak and the deepest hole are located 0.98 and 2.06 Å from the Ba2 and B atoms, respectively.

## Supplementary Material

Crystal structure: contains datablock(s) I. DOI: 10.1107/S2056989017001864/wm5348sup1.cif

Structure factors: contains datablock(s) I. DOI: 10.1107/S2056989017001864/wm5348Isup2.hkl

Crystal structure. DOI: 10.1107/S2056989017001864/wm5348sup3.txt

CCDC reference: 1530747

Additional supporting information:  crystallographic information; 3D view; checkCIF report

## Figures and Tables

**Table 1:** Selected geometric parameters (Å, °)

(Pb/Ba)1—O1	2.537 (3)	B—O1	1.318 (5)
Ba2—O1	2.766 (3)	B—O2	1.397 (5)
Ba2—O1^i^	2.810 (3)	B—O2^ii^	1.406 (5)
Ba2—O2	3.030 (3)		
			
O1—B—O2	120.6 (4)	O2—B—O2^ii^	116.8 (4)
O1—B—O2^ii^	122.6 (4)		

Symmetry codes: (i) 
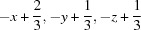
; (ii) 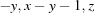.

**Table 2:** Experimental details

Crystal data	
Chemical formula	Ba_8.35_Pb_0.65_(B_3_O_6_)_6_
*M*_r_	2051.83
Crystal system, space group	Trigonal, *R* 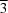
Temperature (K)	296
*a*, *c* (Å)	7.206 (2), 18.653 (11)
*V* (Å^3^)	838.7 (6)
*Z*	1
Radiation type	Mo *K*α
μ (mm^−1^)	13.00
Crystal size (mm)	0.16 × 0.08 × 0.02

Data collection	
Diffractometer	Bruker APEXII CCD
Absorption correction	Numerical (face-indexed using *SADABS*; Bruker, 2000 ▸)
*T*_min_, *T*_max_	0.141, 0.547
No. of measured, independent and observed [*I* > 2σ(*I*)] reflections	1745, 441, 430
*R*_int_	0.024
(sin θ/λ)_max_ (Å^−1^)	0.652

Refinement	
*R*[*F* ^2^ > 2σ(*F* ^2^)], *wR*(*F* ^2^), *S*	0.019, 0.048, 1.23
No. of reflections	441
No. of parameters	35
Δρ_max_, Δρ_min_ (e Å^−3^)	0.64, −0.74

Computer programs: *APEX2* and *SAINT* (Bruker, 2000 ▸), *SHELXS97*, *SHELXL97* and *SHELXTL* (Sheldrick, 2008 ▸) and *DIAMOND* (Brandenburg, 2006 ▸).

## supplementary crystallographic information

### Crystal data

**Table:**

Ba_8.35_Pb_0.65_(B_3_O_6_)_6_	*D*_x_ = 4.062 Mg m^−^^3^
*M**_r_* = 2051.83	Mo *K*α radiation, λ = 0.71073 Å
Trigonal, *R*3	Cell parameters from 1161 reflections
Hall symbol: -R 3	θ = 3.3–27.6°
*a* = 7.206 (2) Å	µ = 13.00 mm^−^^1^
*c* = 18.653 (11) Å	*T* = 296 K
*V* = 838.7 (6) Å^3^	Plate, colourless
*Z* = 1	0.16 × 0.08 × 0.02 mm
*F*(000) = 899	

### Data collection

**Table:**

Bruker APEXII CCD diffractometer	441 independent reflections
Radiation source: fine-focus sealed tube	430 reflections with *I* > 2σ(*I*)
Graphite monochromator	*R*_int_ = 0.024
phi and ω scans	θ_max_ = 27.6°, θ_min_ = 3.3°
Absorption correction: numerical (face-indexed using *SADABS*; Bruker, 2000)	*h* = −8→9
*T*_min_ = 0.141, *T*_max_ = 0.547	*k* = −9→9
1745 measured reflections	*l* = −14→23

### Refinement

**Table:**

Refinement on *F*^2^	Primary atom site location: structure-invariant direct methods
Least-squares matrix: full	Secondary atom site location: difference Fourier map
*R*[*F*^2^ > 2σ(*F*^2^)] = 0.019	*w* = 1/[σ^2^(*F*_o_^2^) + (0.0178*P*)^2^ + 5.852*P*] where *P* = (*F*_o_^2^ + 2*F*_c_^2^)/3
*wR*(*F*^2^) = 0.048	(Δ/σ)_max_ < 0.001
*S* = 1.23	Δρ_max_ = 0.64 e Å^−^^3^
441 reflections	Δρ_min_ = −0.74 e Å^−^^3^
35 parameters	Extinction correction: SHELXL97 (Sheldrick, 2008), Fc^*^=kFc[1+0.001xFc^2^λ^3^/sin(2θ)]^-1/4^
0 restraints	Extinction coefficient: 0.00125 (17)

### Special details

**Table:**

Geometry. All esds (except the esd in the dihedral angle between two l.s. planes) are estimated using the full covariance matrix. The cell esds are taken into account individually in the estimation of esds in distances, angles and torsion angles; correlations between esds in cell parameters are only used when they are defined by crystal symmetry. An approximate (isotropic) treatment of cell esds is used for estimating esds involving l.s. planes.
Refinement. Refinement of F^2^ against ALL reflections. The weighted R-factor wR and goodness of fit S are based on F^2^, conventional R-factors R are based on F, with F set to zero for negative F^2^. The threshold expression of F^2^ > 2sigma(F^2^) is used only for calculating R-factors(gt) etc. and is not relevant to the choice of reflections for refinement. R-factors based on F^2^ are statistically about twice as large as those based on F, and R- factors based on ALL data will be even larger.

### Fractional atomic coordinates and isotropic or equivalent isotropic displacement parameters (Å^2^)

**Table:**

	*x*	*y*	*z*	*U*_iso_*/*U*_eq_	Occ. (<1)
Pb1	0.0000	0.0000	0.0000	0.0147 (2)	0.216 (7)
Ba1	0.0000	0.0000	0.0000	0.0147 (2)	0.784 (7)
Ba2	0.6667	0.3333	0.12947 (2)	0.01537 (18)	
B	0.2953 (7)	−0.1579 (7)	0.0864 (3)	0.0175 (9)	
O1	0.2633 (5)	0.0063 (4)	0.09165 (18)	0.0218 (6)	
O2	0.5029 (5)	−0.1261 (5)	0.08345 (18)	0.0234 (7)	

### Atomic displacement parameters (Å^2^)

**Table:**

	*U*^11^	*U*^22^	*U*^33^	*U*^12^	*U*^13^	*U*^23^
Pb1	0.0136 (2)	0.0136 (2)	0.0169 (3)	0.00680 (12)	0.000	0.000
Ba1	0.0136 (2)	0.0136 (2)	0.0169 (3)	0.00680 (12)	0.000	0.000
Ba2	0.01176 (19)	0.01176 (19)	0.0226 (3)	0.00588 (9)	0.000	0.000
B	0.019 (2)	0.017 (2)	0.018 (2)	0.0103 (18)	−0.0009 (18)	−0.0002 (17)
O1	0.0185 (14)	0.0159 (13)	0.0320 (17)	0.0093 (12)	−0.0011 (12)	−0.0022 (12)
O2	0.0162 (14)	0.0142 (13)	0.0384 (18)	0.0066 (11)	0.0012 (13)	0.0017 (13)

### Geometric parameters (Å, º)

**Table:**

(Pb/Ba)1—O1^i^	2.537 (3)	Ba2—O1^vii^	2.810 (3)
(Pb/Ba)1—O1^ii^	2.537 (3)	Ba2—O2	3.030 (3)
(Pb/Ba)1—O1	2.537 (3)	Ba2—O2^viii^	3.030 (3)
(Pb/Ba)1—O1^iii^	2.537 (3)	Ba2—O2^ix^	3.030 (3)
(Pb/Ba)1—O1^iv^	2.537 (3)	Ba2—B^viii^	3.296 (5)
(Pb/Ba)1—O1^v^	2.537 (3)	Ba2—B^ix^	3.296 (5)
Pb1—Ba2^vi^	3.803 (2)	Ba2—Pb1^xii^	3.803 (2)
Pb1—Ba2^vii^	3.803 (2)	B—O1	1.318 (5)
Ba2—O1^viii^	2.766 (3)	B—O2	1.397 (5)
Ba2—O1	2.766 (3)	B—O2^xiii^	1.406 (5)
Ba2—O1^ix^	2.766 (3)	O1—Ba2^vii^	2.810 (3)
Ba2—O1^x^	2.810 (3)	O2—B^xiv^	1.406 (5)
Ba2—O1^xi^	2.810 (3)		
			
O1^i^—Pb1—O1^ii^	100.43 (11)	O1^xi^—Ba2—O2^viii^	67.48 (9)
O1^i^—Pb1—O1	180.00 (17)	O1^vii^—Ba2—O2^viii^	135.55 (8)
O1^ii^—Pb1—O1	79.57 (11)	O2—Ba2—O2^viii^	112.31 (6)
O1^i^—Pb1—O1^iii^	79.57 (11)	O1^viii^—Ba2—O2^ix^	147.14 (9)
O1^ii^—Pb1—O1^iii^	180.00 (11)	O1—Ba2—O2^ix^	67.39 (8)
O1—Pb1—O1^iii^	100.43 (11)	O1^ix^—Ba2—O2^ix^	47.75 (8)
O1^i^—Pb1—O1^iv^	79.57 (11)	O1^x^—Ba2—O2^ix^	135.55 (8)
O1^ii^—Pb1—O1^iv^	100.43 (11)	O1^xi^—Ba2—O2^ix^	107.59 (8)
O1—Pb1—O1^iv^	100.43 (11)	O1^vii^—Ba2—O2^ix^	67.48 (9)
O1^iii^—Pb1—O1^iv^	79.57 (11)	O2—Ba2—O2^ix^	112.31 (6)
O1^i^—Pb1—O1^v^	100.43 (11)	O2^viii^—Ba2—O2^ix^	112.31 (6)
O1^ii^—Pb1—O1^v^	79.57 (11)	O1^viii^—Ba2—B^viii^	23.05 (10)
O1—Pb1—O1^v^	79.57 (11)	O1—Ba2—B^viii^	134.00 (10)
O1^iii^—Pb1—O1^v^	100.43 (11)	O1^ix^—Ba2—B^viii^	92.24 (10)
O1^iv^—Pb1—O1^v^	180.00 (17)	O1^x^—Ba2—B^viii^	89.14 (10)
O1^i^—Pb1—Ba2^vi^	47.64 (7)	O1^xi^—Ba2—B^viii^	75.27 (10)
O1^ii^—Pb1—Ba2^vi^	132.36 (7)	O1^vii^—Ba2—B^viii^	144.45 (11)
O1—Pb1—Ba2^vi^	132.36 (7)	O2—Ba2—B^viii^	89.88 (9)
O1^iii^—Pb1—Ba2^vi^	47.64 (7)	O2^viii^—Ba2—B^viii^	25.06 (9)
O1^iv^—Pb1—Ba2^vi^	47.64 (7)	O2^ix^—Ba2—B^viii^	134.51 (10)
O1^v^—Pb1—Ba2^vi^	132.36 (7)	O1^viii^—Ba2—B^ix^	134.00 (10)
O1^i^—Pb1—Ba2^vii^	132.36 (7)	O1—Ba2—B^ix^	92.24 (10)
O1^ii^—Pb1—Ba2^vii^	47.64 (7)	O1^ix^—Ba2—B^ix^	23.05 (10)
O1—Pb1—Ba2^vii^	47.64 (7)	O1^x^—Ba2—B^ix^	144.45 (11)
O1^iii^—Pb1—Ba2^vii^	132.36 (7)	O1^xi^—Ba2—B^ix^	89.14 (10)
O1^iv^—Pb1—Ba2^vii^	132.36 (7)	O1^vii^—Ba2—B^ix^	75.27 (10)
O1^v^—Pb1—Ba2^vii^	47.64 (7)	O2—Ba2—B^ix^	134.51 (10)
Ba2^vi^—Pb1—Ba2^vii^	180.0	O2^viii^—Ba2—B^ix^	89.88 (10)
O1^viii^—Ba2—O1	113.73 (6)	O2^ix^—Ba2—B^ix^	25.06 (9)
O1^viii^—Ba2—O1^ix^	113.73 (6)	B^viii^—Ba2—B^ix^	114.27 (7)
O1—Ba2—O1^ix^	113.73 (6)	O1^viii^—Ba2—Pb1^xii^	104.78 (7)
O1^viii^—Ba2—O1^x^	76.27 (10)	O1—Ba2—Pb1^xii^	104.78 (7)
O1—Ba2—O1^x^	89.15 (12)	O1^ix^—Ba2—Pb1^xii^	104.78 (7)
O1^ix^—Ba2—O1^x^	145.30 (7)	O1^x^—Ba2—Pb1^xii^	41.85 (7)
O1^viii^—Ba2—O1^xi^	89.15 (12)	O1^xi^—Ba2—Pb1^xii^	41.85 (7)
O1—Ba2—O1^xi^	145.30 (7)	O1^vii^—Ba2—Pb1^xii^	41.85 (7)
O1^ix^—Ba2—O1^xi^	76.27 (10)	O2—Ba2—Pb1^xii^	106.46 (7)
O1^x^—Ba2—O1^xi^	70.59 (10)	O2^viii^—Ba2—Pb1^xii^	106.46 (7)
O1^viii^—Ba2—O1^vii^	145.31 (7)	O2^ix^—Ba2—Pb1^xii^	106.46 (7)
O1—Ba2—O1^vii^	76.27 (10)	B^viii^—Ba2—Pb1^xii^	104.10 (8)
O1^ix^—Ba2—O1^vii^	89.15 (12)	B^ix^—Ba2—Pb1^xii^	104.10 (8)
O1^x^—Ba2—O1^vii^	70.59 (10)	O1—B—O2	120.6 (4)
O1^xi^—Ba2—O1^vii^	70.59 (10)	O1—B—O2^xiii^	122.6 (4)
O1^viii^—Ba2—O2	67.39 (8)	O2—B—O2^xiii^	116.8 (4)
O1—Ba2—O2	47.75 (8)	B—O1—Pb1	113.5 (3)
O1^ix^—Ba2—O2	147.14 (9)	B—O1—Ba2	101.7 (3)
O1^x^—Ba2—O2	67.48 (9)	Pb1—O1—Ba2	130.17 (12)
O1^xi^—Ba2—O2	135.55 (8)	B—O1—Ba2^vii^	117.4 (3)
O1^vii^—Ba2—O2	107.59 (8)	Pb1—O1—Ba2^vii^	90.51 (10)
O1^viii^—Ba2—O2^viii^	47.75 (8)	Ba2—O1—Ba2^vii^	103.73 (10)
O1—Ba2—O2^viii^	147.14 (9)	B—O2—B^xiv^	122.9 (4)
O1^ix^—Ba2—O2^viii^	67.39 (8)	B—O2—Ba2	88.2 (2)
O1^x^—Ba2—O2^viii^	107.59 (8)	B^xiv^—O2—Ba2	143.4 (3)

Symmetry codes: (i) −*x*, −*y*, −*z*; (ii) −*y*, *x*−*y*, *z*; (iii) *y*, −*x*+*y*, −*z*; (iv) *x*−*y*, *x*, −*z*; (v) −*x*+*y*, −*x*, *z*; (vi) *x*−2/3, *y*−1/3, *z*−1/3; (vii) −*x*+2/3, −*y*+1/3, −*z*+1/3; (viii) −*y*+1, *x*−*y*, *z*; (ix) −*x*+*y*+1, −*x*+1, *z*; (x) *y*+2/3, −*x*+*y*+1/3, −*z*+1/3; (xi) *x*−*y*+2/3, *x*+1/3, −*z*+1/3; (xii) *x*+2/3, *y*+1/3, *z*+1/3; (xiii) −*y*, *x*−*y*−1, *z*; (xiv) −*x*+*y*+1, −*x*, *z*.

## References

[bb1] Bartwal, K. S., Bhatt, R., Kar, S. & Wadhawan, V. K. (2001). *Mater. Sci. Eng. B*, **85**, 76–79.

[bb2] Brandenburg, K. (2006). *DIAMOND*. Crystal Impact GbR, Bonn, Germany.

[bb3] Bruker (2000). *APEX2*, *SAINT* and *SADABS*. Bruker AXS Inc., Madison, Wisconsin, USA.

[bb4] Chen, C., Wang, Y., Wu, B., Wu, K., Zeng, W. & Yu, L. (1995). *Nature*, **373**, 322–324.

[bb5] Chen, C., Wu, Y., Jiang, A., Wu, B., You, G., Li, R. & Lin, S. (1989). *J. Opt. Soc. Am. B*, **6**, 616–621.

[bb6] Chen, C., Wu, B., Jiang, A. & You, G. (1985). *Sci. Sin.* B**28**, 235–241.

[bb7] Dong, X. Y., Jing, Q., Shi, Y. J., Yang, Z. H., Pan, S. L., Poeppelmeier, K. R., Young, J. & Rondinelli, J. M. (2015). *J. Am. Chem. Soc.* **137**, 9417–9422. 10.1021/jacs.5b05406 26147880

[bb8] Huang, Y., Wu, L., Wu, X., Li, L., Chen, L. & Zhang, Y. (2010). *J. Am. Chem. Soc.* **132**, 12788–12789. 10.1021/ja106066k 20738133

[bb9] Jing, Q., Dong, X. Y., Yang, Z. H. & Pan, S. L. (2015). *Dalton Trans.* **44**, 16818–16823. 10.1039/c5dt02652k 26347088

[bb10] Li, H. Y., Dong, L., Lu, Y., Pan, S. L., Lu, X., Yu, H. W., Wu, H. P., Su, X. & Yang, Z. H. (2014). *J. Alloys Compd.* **615**, 561–565.

[bb11] Liu, L. L., Yang, Y., Jing, Q., Dong, X. Y., Yang, Z. H., Pan, S. L. & Wu, K. (2016). *J. Phys. Chem. C*, **120**, 18763–18770.

[bb12] Liu, L., Zhang, B. B., Zhang, F. F., Pan, S. L., Zhang, F. Y., Zhang, X. W., Dong, X. Y. & Yang, Z. H. (2015). *Dalton Trans.* **44**, 7041–7047. 10.1039/c5dt00641d 25785911

[bb13] Mutailipu, M., Hou, D. W., Zhang, M., Yang, Z. H. & Pan, S. L. (2016). *New J. Chem.* **40**, 6120–6126.

[bb14] Sasaki, T., Mori, Y., Yoshimura, M., Yap, Y. K. & Kamimura, T. (2000). *Mater. Sci. Eng. Rep.* **30**, 1–54.

[bb15] Sheldrick, G. M. (2008). *Acta Cryst.* A**64**, 112–122. 10.1107/S0108767307043930 18156677

[bb16] Tang, X. L., Feng, D. X., Wan, S. M., Kang, L., Zhang, B. & Lin, Z. S. (2015). *Mater. Chem. Phys.* **163**, 501–506.

[bb17] Wu, H. P., Pan, S. L., Jia, D. Z., Chen, Z. H. & Yu, H. W. (2012). *Chem. Lett.* **41**, 812–813.

[bb18] Wu, S., Wang, G., Xie, J., Wu, X., Zhang, Y. & Lin, X. (2002). *J. Cryst. Growth*, **245**, 84–86.

[bb19] Zhang, G., Liu, Z., Zhang, J., Fan, F., Liu, Y. & Fu, P. (2009). *Cryst. Growth Des.* **9**, 3137–3141.

[bb20] Zhang, F. F., Zhang, F. Y., Lei, B. H., Yang, Z. H. & Pan, S. L. (2016). *J. Phys. Chem. C*, **120**, 12757–12764.

[bb21] Zhang, H., Zhang, M., Pan, S. L., Yang, Z., Wang, Z., Bian, Q., Hou, X., Yu, H., Zhang, F., Wu, K., Yang, F., Peng, Q., Xu, Z., Chang, K. B. & Poeppelmeier, K. R. (2015). *Cryst. Growth Des.* **15**, 523–529.

